# Nutritional Quality of Breakfast Cereals on the French, Belgian and Luxembourg Markets: Which Cereals for Children?

**DOI:** 10.3390/nu16162701

**Published:** 2024-08-14

**Authors:** Martine Robert, Françoise Martin, Annick Xhonneux, Françoise Mosser, Elisabeth Favre, Celine Richonnet

**Affiliations:** Club Européen des Diététiciens de l’Enfance (CEDE), Esplanade, 17-7800 Ath, Belgium

**Keywords:** breakfast cereals, children, food marketing, nutritional quality, Nutri-Score, food composition, nutrition labelling, sugar, ultra-processed foods

## Abstract

**Objective**: Analyse the breakfast cereal market to help to help healthcare professionals to guide parents in choosing healthy products for their children. **Study design**: Observational study of the breakfast cereals available in the biggest supermarkets, discount stores and organic chains in France, Belgium and Luxembourg. **Methods**: An analysis of nutritional qualities using three indicators: Nutri-Score (initial and modified version), WHO Europe nutrient profile model, and Nova. **Results**: 645 products were listed; 559 excluding duplicates. A total of 28.8% are marketed to children and make up the group of “children’s” cereals, 62.1% of cereals are Muesli, Oats and other cereal flakes (MOCF), and 54.9% are “organic”. The study shows that “children’s” cereals have a poorer nutritional profile: a higher proportion of Nutri-Score D, higher sugar content, lower fibre content, less conformity with the WHO Europe nutrient profile model and a higher proportion ofultra-processed. On the other hand, MOCF and “organic” products generally have a better nutritional profile: less sugar, more fibre, more Nutri-Score A, less Nutri-Score D and fewer ultra-processed products. **Conclusions**: Parents should therefore opt for cereals that do not bear any reference to children on the packaging.

## 1. Introduction

An emblematic choice for children’s breakfasts, breakfast cereals have become increasingly popular and now have high penetration rates of 54.4% among 6- to 17-year-olds in France [1].

Breakfast, the first meal of the day, plays a critical role in children’s energy balance and dietary regulation [2]. Several studies have shown a link with excess weight and obesity [3]. The systematic review by Monzani et al. including 286,804 children and adolescents in 33 countries [4] demonstrated that eating breakfast is associated with a lower BMI in children and adolescents, as well as with better nutritional intake [5] and better cognitive status [6] in school-age children.

There are high rates of obesity in Europe, with 29.5% of 5- to 9-year-olds and 24.9% of 10- to 19-year-olds overweight, including 11.6% and 7.1% obese subjects, respectively [7]. This is increasing significantly according to school surveys [8], marking an acceleration in growth since the pandemic.

As a professional association of paediatric dietitians, the CEDE (Club Européen des Diététiciens de l’Enfance), we wanted to assess the nutritional quality and extent of processing of the breakfast cereals available on the French, Belgian and Luxembourg markets in order to support our healthcare professionals members to identify which cereals are best suited to children. To do so, we analysed the nutritional profile using three indicators: Nutri-Score, WHO Europe nutrient profile model, and the Nova classification system [9].

## 2. Materials and Methods

For each country, the main supermarket, discount store and specialist organic shop retailer in terms of volume was selected based on market share: Leclerc, Lidl, Biocoop in France; Colruyt, Aldi, BioPlanet in Belgium; Cactus, Aldi, Naturata in Luxembourg. All the ready-to-eat and ready-to-prepare breakfast cereal products were collected exhaustively in each of these chains between 3 and 25 January 2022. Each pack was photographed in its entirety and all the relevant data were then processed. Any data entered manually by one of the authors were checked by the other operators.

### 2.1. Children’s Marketing

Marketing elements aimed at children were identified using the International Network for Food and Obesity/NCDs Research Monitoring and Action Support (INFORMAS) protocol [10]: childlike drawings, licences (e.g., Barbie^®^), games on the packaging or encouragement to play online that call on children’s cognitive abilities, prizes, and references to children or their attributes (schoolbag, skateboard, hopscotch, etc.) drawn or mentioned in the text.

### 2.2. Nutri-Score

France’s 2019–2024 National Nutrition and Health Programme (PNNS) recommends using the Nutri-Score indicator to select foods that are to be encouraged, and reducing consumption of Nutri-Score D and E products, particularly among children [11]. We recorded the Nutri-Score when available on front-of-pack and calculated those that were not available on packaging from the nutritional values in the table of nutrition facts. We used the initial algorithm and its modified version, adopted on 26 July 2022 and implemented in 2024, using the spreadsheet provided by Santé Publique France [12].

Energy, total sugar, saturated fat, and sodium score negative points, while fruits, vegetables, protein, and fibre score positive points. The algorithm on which Nutri-Score is based is on a continuous and discrete scale ranging from +40 (least healthy) to −15 (most healthy).

### 2.3. WHO Europe Nutrient Profile Model

The Regional Office for Europe of the World Health Organization (WHO) developed a model nutritional profile in 2015, and updated it in 2023, with the aim of restricting food marketing and advertising to children [13]. It can be considered a benchmark in terms of nutrition and the composition of children’s food. We compared the nutrition facts in the nutrition panels on the packaging with the breakfast cereals category thresholds: no more than 17 g of fat, 12.5 g of total sugars and 0.5 g of sodium.

### 2.4. Degree of Food Processing

The 2019–2024 National Nutrition and Health Programme set the target of halting growth in the consumption of ultra-processed foods (UPFs) (according to the Nova classification) and reducing their consumption by 20% for the population as a whole, including children [11].

The Nova classification was used to categorise the foods included in the study into unprocessed or minimally processed (Nova 1), processed (Nova 3) and ultra-processed (Nova 4) after analysing each component in the list of ingredients and noting the presence of markers: ultra-processed ingredients derived from extraction (gluten, lactose, caseins, etc.) and secondary processing (glucose–fructose syrup, hydrogenated fats, etc.); cosmetic additives (colourings, emulsifiers, melting salts, gelling and texturing agents, sweeteners, etc.) and flavourings (natural or otherwise) [9].

### 2.5. Statistical Analysis

Results are expressed as mean and standard deviation (SD) for quantitative data and as median and quartiles for skewed data. Frequency tables (number, percent) are used for categorical data. Logistic regression analysis was used to assess the relationship between a binary characteristic (e.g., MOCF: yes vs. no) and several covariates (sugar, fibre, saturated fatty acids, quantity of cereals). Results are then expressed as regression coefficients with standard error (SE) or as odds ratio (OR) with a 95% confidence interval (95%CI). Results are considered significant at the 5% critical level (*p* < 0.05). All calculations were performed with SAS version 9.4.

## 3. Results

### 3.1. Sample Characteristics (Supplemental Table S1)

A total of 645 products were found in the 9 chains visited: 56.7% in supermarkets, 11.8% in discount stores and 31.5% in organic shops. The products were classified based on their legal sales denomination according to the classification developed by Oqali [14]. The main categories represented are oats and mueslis (57.5%), followed by filled, extruded or puffed cereals (29.0%) and flakes (12.2%). In discount stores, filled, extruded or puffed cereals (43.4%) represent the majority, ahead of oats and mueslis (38.2%). In organic shops, oats and mueslis are over-represented (78.3%).

More than half the sample is organic (51.6%; 333 references), 61.0% of which comes from organic shops. We note that 44.8% of the products included displayed their Nutri-Score: 61.7% in supermarkets, 65.8% in discount stores and only 6.4% in organic shops.

**Figure 1:**
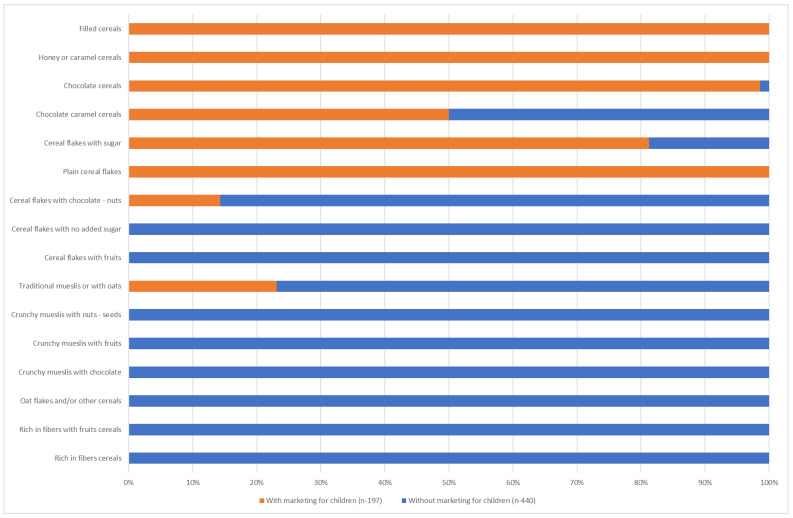
Percentage of cereals with marketing targeting children.

The majority of chocolate cereals, chocolate and caramel cereals, honey or caramel cereals, filled cereals and sugared cereal flakes have marketing elements aimed at children (68% to 100%) (Figure 1). We will therefore consider cereals from these 5 families as “children’s” cereals in the rest of the study (*n* = 206, i.e., 31.9%). The majority (66.0%) of these products are from supermarkets, 18.0% from discount stores and 16.0% from specialist organic shops. Only 27.7% of these products are organic, and most of them (57.9%) are sold in organic shops.

We found 66 identical products, mainly (45) children’s products, in different chains and even countries. For all subsequent analyses, duplicates were removed, reducing the sample size to 559 (Supplemental Table S1), of which 161 (28.8%) were “children’s” cereals, 347 (62.1%) were MOCF and 307 (54.9%) were labelled “organic”. We also note that 29.2% of “children’s” cereals were labelled “organic”.

### 3.2. Nutritional Profile (Figure 2 and Figure 3, Table 1 and Table S2)

Studying the nutritional profile of the sample revealed major differences among the nutrients studied. Total sugar content varies from 0 to 40.0 g per 100 g depending on the category. Reading the list of ingredients revealed that 78.2% of cereals contain added sugars (sucrose, glucose syrup, honey, etc.). The fibre content varies from 0 to 29.0 g per 100 g, and the fat content varies from 0.5 to 51.0 g per 100 g with 0 to 16.4 g per 100 g of saturated fatty acids (SFA).

Statistical analyses (Table 1) show that the “children’s” cereals contain significantly more sugar, less SFA and less fibre than the rest of the sample. 

**Table 1:** Associations between cereal characteristics and their composition.

Composition	MOCF	“Children’s” Cereals	“Organic” Cereals
Intercept	−0.85 ± 0.456	−0.24 ± 0.557	0.91 ± 0.393
SFA	0.52 ± 0.075 *	−0.73 ± 0.109 *	0.07 ± 0.038
Sugar	−0.13 ± 0.017 *	0.23 ± 0.025 *	−0.09 ± 0.013 *
Fibre	0.34 ± 0.049 *	−0.50 ± 0.068 *	0.08 ± 0.034 *

* significant; the results are expressed in terms of (logistic) regression coefficients and standard errors; a positive (negative) coefficient corresponds to an increase (decrease) in the product’s composition compared with the others.

**Figure 2:**
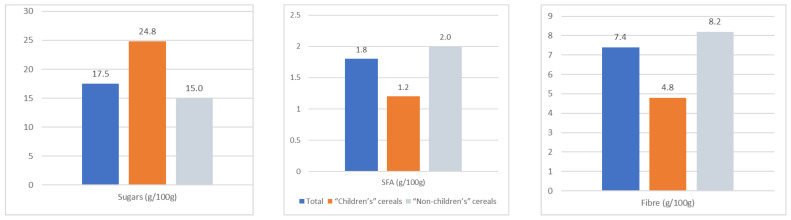
Comparison of the nutritional profile of “children’s” cereals with other cereals and the total sample.

The median sugar content of “children’s” cereals is 24.8 g per 100 g, compared with 15.0 g per 100 g for other cereals (*p* < 0.0001) (Figure 2). These sugars are added sugars, i.e., free sugars, as the products do not contain any dairy products or fruit, which are sources of intrinsic sugars.

**Figure 3:**
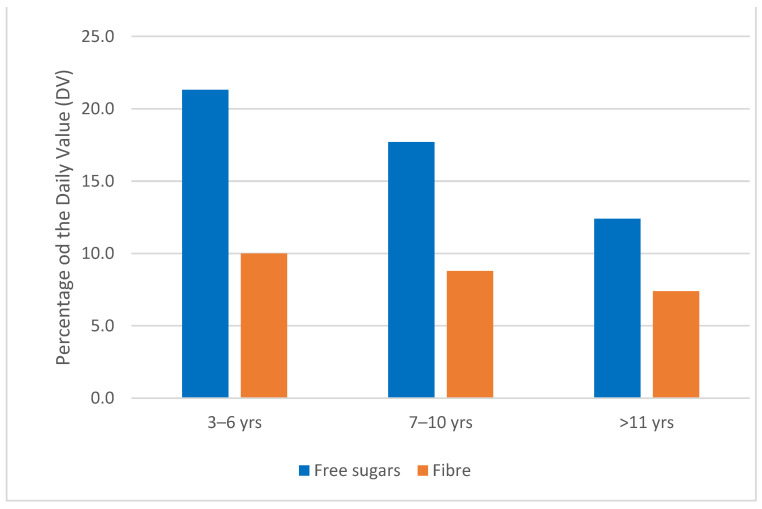
Percentage of recommended intake of free sugars and fibre for a 30 g portion of “children’s” cereals (*n* = 161).

A large number (45%) of “children’s” cereals recommend a 30 g portion. This portion size provides between 12.4% and 21.3% of the WHO’s recommended sugar intake for children, depending on their age (Figure 3).

“Children’s” cereals provide significantly less fibre: median 4.8 g per 100 g compared with 8.2 g per 100 g for other cereals (Figure 2). Based on a 30 g serving, “children’s” cereals never provide more than 10% of the nutritional fibre guidelines defined by EFSA [15] (Figure 3), and generally fall below 10% for 11- to 14-year-olds.

“Children’s” cereals are three times lower in fat (3.8 g per 100 g compared with 12 g per 100 g) despite the high prevalence of chocolate versions. The SFA content in children’s cereals is significantly lower than that of other cereals, with a median of 1.2 g compared with 2.0 g (Supplemental Table S2).

The same logistic regression analyses show that MOCFs contain significantly less sugar but more SFAs and fibre than the rest of the sample (Table 1). This category contains significantly less sugar than the other categories (median: 15 g per 100 g vs. 22.4 g per 100 g). Oats and flakes with no added sugar have lower contents of 1.1 and 0.5 g per 100 g, respectively. The median fat content of MOCFs is 13.0 g per 100 g with 2.0 g per 100 g of SFA. Crunchy mueslis have the highest fat content, with a median of 16 g per 100 g. MOCFs provide significantly more fibre than other cereals (8.2 vs. 6.2 g per 100 g).

Finally, the logistic regression analysis shows that the “organic” cereals contain significantly more fibre and less sugar (median content: 14.0 g per 100 g vs. 20.9 g per 100 g), but do not differ in terms of SFAs.

### 3.3. Nutri-Score (Supplemental Tables S3 and S4)

**Figure 4:**
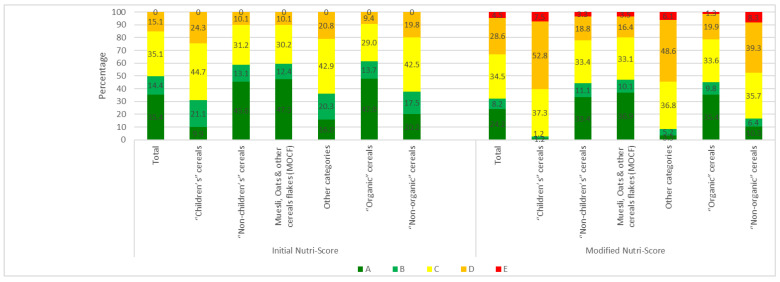
Distribution of Nutri-Scores for “children’s” cereals (*n* = 161), MO cereals (*n* = 347), “organic” cereals (*n* = 307) and the total sample (*n* = 559) with the initial algorithm (**left**) and the future algorithm (**right**).

In the sample as a whole (Figure 4), the majority were Nutri-Score A (35.4%) and C (35.1%). The modified Nutri-Score is more discriminating for breakfast cereals, with only 24.2% A scores, 8.2% B scores, 34.5% C scores, and D scores rising to 28.6%. In addition, E scores appear and represent 4.5%.

With the initial Nutri-Score, “children’s” cereals have significantly less Nutri-Score A, and more Nutri-Score B, C and D than other cereals (OR = 3.6, CI95%: 2.6–5.2). The modified version of the Nutri-Score further accentuates the difference with other cereals (OR = 6.5, CI95%: 4.5–9.4). For children’s cereals, Nutri-Scores A and B virtually disappear, and the E score, which was totally absent from the initial version, appears.

In the initial version of the Nutri-Score, “organic” cereals have significantly more A scores (47.9%) and fewer D scores (9.5%) (Figure 4) than conventional cereals, with 20.2% and 19.8%, respectively (*p* < 0.0001) (Supplemental Table S3). The modified version of the Nutri-Score algorithm exacerbates the nutritional superiority of “organic” cereals with fewer Nutri-Score D scores (19.9% compared with 39.3% for “non-organic” cereals) and more A scores (35.5% compared with 10.3% for “non-organic” cereals) (Figure 4). Furthermore, with the modified Nutri-Score, the E score is present in 1.3% of “organic” cereals (Figure 4) compared with 8.3% of “non-organic cereals” (Supplemental Table S3). MOCFs score more favourably than other cereals. However, the modified Nutri-Score downgrades MOCFs in the same way as all the other categories, with the appearance of an E score.

**Figure 5:**
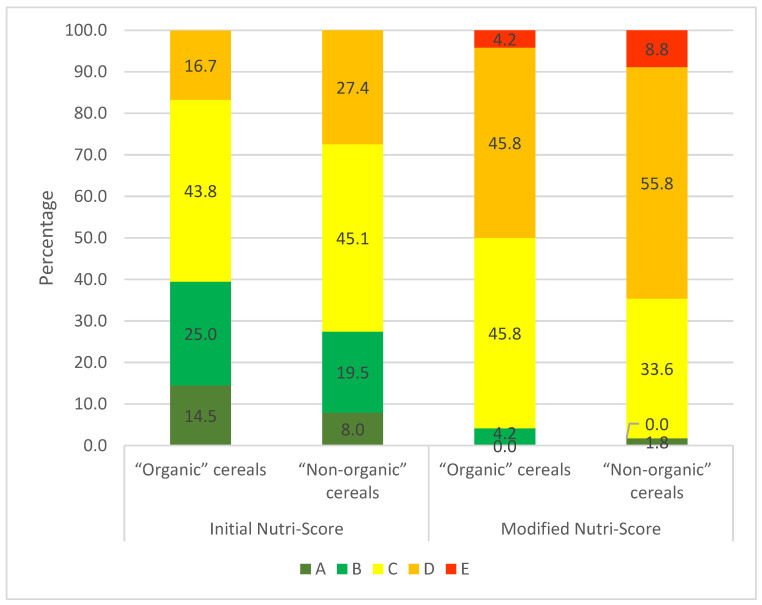
Nutri-Score distribution for “organic” (*n* = 48) and “non-organic” (*n* = 113) “children’s” cereals with the initial algorithm (**left**) and the modified algorithm (**right**).

For “children’s” cereals, we also see better scores for “organic” products, but to a lesser extent: 14.6% vs. 8.0% for score A in the initial Nutri-Score and 0% vs. 1.8% of Nutri-Score A for the modified Nutri-Score (Figure 5). The percentage of D scores is lower with the initial Nutri-Score for “organic” products: 16.7% vs. 27.4%. This observation also applies to the modified Nutri-Score: 45.8% vs. 55.8%. The same applies to the E score observed only in the modified version of the Nutri-Score: 4.2% for “organic” products compared with 8.8% for other products.

### 3.4. Compliance with the WHO Europe Nutrient Profile Model 

For the sample as a whole, only a quarter of the products met the WHO criteria (24.2%) (Table 2). Of the 161 “children’s” products, 4.4% comply with WHO nutritional criteria and are suitable for children. This figure is well below that of the “non-children” categories, which have a compliance rate of 32.2%. Of the 154 non-compliant “children’s” products, 148 (96.1%) did not meet the total sugar criterion (≤12.5 g per 100 g).

In the MOCF category, compliance with the WHO standard was significantly higher (32% of products) (*p* < 0.0001). “Organic” products are more compliant with WHO criteria than “non-organic” products (33.9% compared with 12.3%). The proportion of products meeting WHO criteria is highest in organic shops (33.5%) and lowest in discount stores (12.5%) and supermarkets (20.9%). None of the 49 “organic” “children’s” cereals comply with WHO criteria.

### 3.5. Degree of Transformation

**Figure 6:**
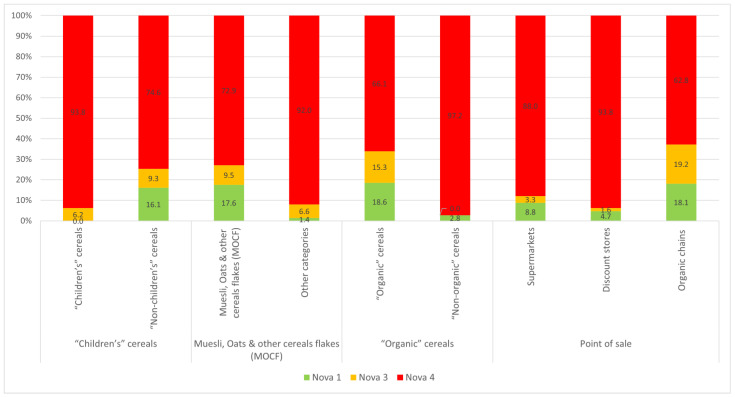
Distribution of Nova scores for all cereals and the various points of sale.

Analysing the ingredient lists reveals that breakfast cereals are extremely “ultra-processed”: 80.1% of the sample is classified as Nova 4 (UPFs), 8.4% are transformed (Nova 3) and only 11.4% are not or slightly transformed (Nova 1) (Figure 6).

“Children’s” cereals are particularly ultra-processed: 93.8% compared with 74.6% for the rest of cereals. No products intended for children meet the Nova 1 score criteria. MOCFs are less ultra-processed (72.1%) than other cereals (92.0%). “Non-organic” cereals are more ultra-processed (97.2%) than “organic” cereals (66.1%). In addition to the ingredient lists, the processing methods, when specified, also reveal more ultra-processing. “Children’s” cereals include 52 extruded cereals, 40 of which are filled and 19 puffed.

When we analyse the points of sale, organic shops contain the most unprocessed/lightly processed products (18.1%), compared to discount stores with 4.7% and supermarkets with 9.8% (Figure 6). “Children’s” cereals have longer ingredient lists (median: 14 elements compared with 12 for other cereals). They also contain more additives (median: 1.6 against 0.8) with a maximum of 7 (Supplemental Table S2). The MOCF category has fewer ingredients (median: 12 elements compared with 14) and fewer additives (0.7 on average compared with 1.5) than other cereals. There are fewer ingredients in “organic” products (median: 11 elements compared with 17) and virtually no additives (0.4 on average compared with 1.7) compared with “non-organic” cereals.

## 4. Discussion

The aim of this study was to analyse which breakfast cereals are best suited to children from the entire range on offer on the French, Belgian and Luxembourg markets. 

The breakfast cereals analysed in this study are almost systematically sweet (78.2%). This proportion rises to 98.1% in “children’s” cereals, which was also observed in the cross-sectional analysis of the nutritional content of 636 “children’s” cereals carried out in Australia, Canada, New Zealand, the United Kingdom and the United States [16]. In this study, 89.7% of “children’s” cereals sold in the United States contained more than 20 g of sugar per 100 g, compared with 74.5% in the United Kingdom. These figures are consistent with our results: in our sample, “children’s” cereals are the only ones to exceed 20 g per 100 g, with a median of 24.8 g per 100 g compared with 15.0 g per 100 g for other cereals. With such high levels, it is worrying that a 30 g bowl provides between 18% and 21% of the recommended daily intake of free sugars for children under 11, especially as the quantities consumed in fact seem to exceed the recommended portion size of 30 g [17]. This high intake of added sugar is a cause for concern in children, as it has been implicated in the prevalence of excess weight and obesity, habituation to sweet tastes and the development of tooth decay [18,19].

“Children’s” cereals contain three times less total fat than other cereals, at 3.8 g per 100 g and 12 g per 100 g, respectively. This observation is in line with the results of a Canadian study of 262 breakfast cereals, with values of 3.6 and 7.2 g [20]. As in the Canadian study (average 0.7 g per 100 g), the SFA content of “children’s” cereals is low, ranging from 0.2 g per 100 g for flakes with sugar to 3.6 g for filled cereals. The use of chocolate in the recipe raises the SFA content of the richest children’s cereals (6.7 g per 100 g).

The importance of fibre’s beneficial role in children’s diets is well established [21,22]. Fibre in breakfast cereals is essential to influence the post-prandial glycaemic response and satiety [23], in particular by acting on gastric emptying [24]. In our study, “children’s” cereals contained less fibre, averaging 4.8 g per 100 g compared with 8.2 g per 100 g. For a 30 g portion, this represents an intake of no more than 10% of the fibre recommendations for children, and even 7.5% for children aged 11 and over. Manufacturers’ efforts to reformulate should be encouraged in order to improve the wholegrain and fibre composition of cereals intended for children [25]. To optimise children’s fibre intake, the recommendation could be to opt for breakfast cereals that are less processed and richer in fibre, such as mueslis with oats and plain cereal oats (8.4 g per 100 g), by choosing wholegrain products with the highest proportion of cereals in the list of ingredients. However, the “high fibre” categories of cereals (above 20 g per 100 g) are not suitable for children’s diets, as they could lead to excess fibre and cause digestive problems and malabsorption of micronutrients [24].

The inappropriate nutritional profile of “children’s” cereals seen in our study has also been observed in other studies [26,27], with “children’s” cereals that are higher in energy, sugars and salt and lower in protein and fibre [28].

Less than 42.0% of the assessed products display their Nutri-Score, which is consistent with the results of our first study [29]. It is used very little in organic shops (present on 6.4% of packaging).

The PNNS recommends the consumption of Nutri-Score A, B and C products [11]. In our study, 75.8% of “children’s” cereals currently meet this criterion, or 39.8% with the modified Nutri-Score, which has stricter criteria on sugar and fibre. Products with a D and E score are mainly chocolate and filled cereals, which are typical in the “children’s” aisle. Similarly, the French [30] and Belgian [31] surveys show that the majority of cereals targeting children are the least balanced. Adding the Nutri-Score to packaging encouraged cereal manufacturers to reduce their sugar content by 5% and their sodium content by 20%, while at the same time increasing their fibre and protein content [31]. We may hope that the modified, stricter Nutri-Score will encourage manufacturers to further improve their products’ composition.

In our sample, 95.4% of “children’s” cereals did not comply with WHO criteria, compared with 67.8% for other cereals, which seems to show that their nutritional composition is of poorer quality and that they are less suitable for the children for whom they are intended (Table 2). Almost all of “children’s” cereals (96.1%) exceed the permitted level of total sugars. This result is in line with those of numerous studies [32,33] which conclude that they are richer in sugars and poorer in fibre. According to a study carried out in Mexico on 3755 adults [34], even if parents are aware of this unsuitable nutritional profile, when buying breakfast cereals they allow themselves to be influenced by their children, who are themselves influenced by the marketing techniques on the packaging.

Several studies have shown that children and adolescents are the main consumers of UPFs [32,35], providing 45.5% of children’s calorie intake in France [36] and 33.3% in Belgium [37]. According to previous studies, the proportion of UPFs in the diet is correlated with its quality, particularly in children [33,38]. In addition to nutritional deficiencies, some studies suggest that the high consumption of UPFs has a negative impact on children and adolescents’ academic ability [39], as well as a correlation with obesity and adiposity [40,41], dyslipidaemia [42,43], tooth decay [44], allergies, eczema and asthma [45] and metabolic syndrome [46] in children. The breakfast cereals analysed in our study are extremely ultra-processed (80.1%). “Children’s” cereals are particularly ultra-processed (93.8%), especially if they are not organic (100%), due to the use of cosmetic additives, flavourings and glucose syrup. Breakfast cereals are often among the top 10 contributors to energy intake from UPFs in children [36,47,48]. These ultra-processed “children’s” products are characterised by a longer list of ingredients and more additives and sugars, as found in the Morales study [49]. Almost half (44.1%) of the “children’s” cereals for which the process is specified are manufactured using ultra-processing techniques. These manufacturing processes can induce changes linked to the matrix effect, in particular on cereals’ metabolic effects, such as their glycaemic index (GI) and their satietogenic effect [50]. These processes can generate a highly gelatinised starch [49,50] and reduce the amylose–amylopectin ratio, which increases the GI [23]. As such, the more elaborate the processing, the higher the GI, as the cereals’ initial structure is destructured [51]. Products that do not target children seem to be more suitable because they are less ultra-processed, as long as we avoid filled or extruded forms and opt for minimally processed options (Muesli, Oats and other cereal flakes (MOCF)).

This study reveals differences in favour of the nutritional quality of “organic” cereals: less sugar, more fibre, more Nutri-Score A, fewer Nutri-Score D and fewer Nova 4 (UPFs). In 2021, an American study [52] comparing 8240 “organic” foods with 72,205 pre-packaged conventional foods showed that the overall nutritional quality of “organic” foods is superior to that of conventional foods.

However, a previous American study [53] (2013) comparing the nutritional scores (NuVal) of 723 conventional breakfast cereals with those of 106 “organic” cereals showed no significant difference between the two groups. Similarly, in 2020, an Italian team [54] concluded that pre-packaged “organic” products were generally no more nutritious than conventional products. The results of our study seem to point towards better overall nutritional quality for “organic” breakfast cereals. The difference in favour of “organic” products is significantly reduced for “children’s” products. Choosing “organic” breakfast cereals increases the chances of having a WHO-compliant product. However, due to excessive sugar content, chances remain low, with only 3 out of 10 “organic” products compliant. Choosing “organic” cereals in an organic shop increases the chances of having a product that complies with WHO criteria. This can be explained by the abundance of MOCF products (79.2% compared with 55.7% in supermarkets and 42.2% of the discount stores’ offer), which are more in line with WHO criteria. “Organic” cereals are less ultra-processed (66.7% vs. 97.1% for conventional cereals), but proportions are still high.

### Strengths and Limitations

This study is limited to the chosen shop chains and does not represent the entire breakfast cereal market in the three selected countries. There is a risk of retranscription with manual data entry, which we reduced by systematically having another operator check the data, and then re-checking any data that changed, making the data more reliable. The manual analysis of the ingredient lists to define the level of processing of each product according to the Nova classification is one of our study’s strengths. To limit errors, we were supported by the research teams who created this classification.

## 5. Conclusions

Breakfast cereals have become an essential part of children’s breakfasts. This study shows that few breakfast cereals meet the nutritional criteria studied. In particular, cereals that target children in France, Belgium and Luxembourg have a poorer nutritional profile than the other cereals on the market, with a higher proportion of Nutri-Score D, a higher sugar content, lower fibre content, less conformity with the WHO model and a higher level of ultra-processing.

Parents who are keen to ensure a balanced diet for their children should therefore opt for cereals that do not bear any reference to children on the packaging.

It is best to choose Nutri-Score A, B or C cereals, which can be seen on the packaging or assessed using a mobile app, and limit the choice to cereals with no more than 17 g of fat, 12.5 g of total sugars and 0.5 g of sodium, fitting with the WHO criteria. When reading the list of ingredients, it is best to opt for products containing more cereals and less sugar, and to keep the ingredient list as short as possible, avoiding unfamiliar ingredients not found in home cooking. Cereals in the form of flakes or oats, which are less processed, are to be preferred. If all the above criteria are met, “organic” products can then be privileged.

Finally, breakfast cereals should be consumed alternately with other breakfast compositions, particularly those that are bread-based.

## Figures and Tables

**Table 2:** Cereal compliance with the WHO Europe nutrient profile model nutritional criteria.

	*n*	WHO Compliant (%)	WHO Non-Compliant (%)
Total sample	559	24.2	75.8
“Children’s” cereals	161	4.4	95.6
Muesli, Oats and other cereal flakes (MOCF)	347	32.0	68.0
“Organic” cereals	307	33.9	77.1

## Data Availability

Data are contained within the article and Supplementary Materials.

## Supplementary Materials

The following supporting information can be downloaded at https://www.mdpi.com/article/10.3390/nu16162701/s1; Table S1: Sample characteristics; Table S2: Nutritional profile of different cereal categories; Table S3: Associations between the Initial Nutri-Score and cereal characteristics; Table S4: Associations between the Modified Nutri-Score and cereal characteristics.
