# Supplementary material for: Nutritional Quality of Breakfast Cereals on the French, Belgian and Luxembourg Markets: Which Cereals for Children?
Source: Nutrients. 2024 Aug 14;16(16):2701. doi: 10.3390/nu16162701 (PMC11357527; doi:10.3390/nu16162701)
Supplement: Supplementary file 1 [file nutrients-16-02701-s001.zip › nutrients-3136414-supplementary.pdf]

Table S1: Sample characteristics

Variable	All products (N=645)	Different products (N=559)
Type of cereals		
OAT AND/OR OTHER CEREALS FLAKES	50 (7.8)	48 (8.6)
TRADITIONAL MUESLIS	99 (15.3)	94 (16.8)
CRUNCHY MUESLIS WITH CHOCOLATE	86 (13.3)	77 (13.8)
CRUNCHY MUESLIS WITH FRUITS	67 (10.4)	66 (11.8)
CRUNCHY MUESLIS WITH NUTS	68 (10.5)	62 (11.1)
PLAIN CEREAL FLAKES	13 (2.0)	9 (1.6)
CEREAL FLAKES WITH FRUITS	6 (0.9)	6 (1.1)
CEREAL FLAKES WITH CHOCOLATE - NUTS	31 (4.8)	22 (3.9)
CEREAL FLAKES WITH NO ADDED SUGAR	10 (1.6)	8 (1.4)
CEREAL FLAKES WITH SUGAR	19 (2.9)	14 (2.5)
HIGH-FIBRE CEREALS	7 (1.1)	5 (0.9)
HIGH-FIBRE CEREALS WITH FRUITS	2 (0.3)	1 (0.2)
HONEY OR CARAMEL CEREALS	56 (8.7)	39 (7.0)
CHOCOLATE CEREALS	73 (11.3)	59 (10.6)
CHOCOLATE CARAMEL CEREALS	5 (0.8)	4 (0.7)
FILLED CEREALS	53 (8.2)	45 (8.1)
Country		
France	265 (41.1)	258 (46.2)
Belgium	193 (29.9)	138 (24.7)
Luxembourg	187 (29.0)	163 (29.2)
Type of shop		
Supermarkets	366 (56.7)	307 (54.9)
Discount stores	76 (11.8)	64 (11.4)
Organic	203 (31.5)	188 (33.6)
Muesli, Oats & other cereals flakes (MOCF) vs. others		
Muesli, Oats & other cereals flakes (MOCF)	370 (57.4)	347 (62.1)
Other	275 (42.6)	212 (37.9)
Cereals for children		
Yes	206 (31.9)	161 (28.8)
No	439 (68.1)	398 (71.2)
Organic product		
Yes	333 (51.6)	307 (54.9)
No	312 (48.4)	252 (45.1)
INITIAL NUTRI-SCORE		
A	216 (33.5)	198 (35.4)
B	97 (15.0)	86 (15.4)
C	236 (36.6)	196 (35.1)
D	96 (14.9)	79 (14.1)
MODIFIED NUTRI-SCORE		
A	144 (22.3)	135 (24.2)
B	47 (7.3)	46 (8.2)
C	219 (34.0)	193 (34.5)
D	202 (31.3)	160 (28.6)
E	33 (5.1)	25 (4.5)

Variable	All products (N=645)	Different products (N=559)
Nova		
1	67 (10.4)	64 (11.4)
3	50 (7.8)	47 (8.4)
4	528 (81.9)	448 (80.1)
Nova		
1 and 3	117 (18.1)	111 (19.9)
4	528 (81.9)	448 (80.1)
WHO 2023 COMPLIANCE		
Yes	150 (23.3)	135 (24.2)
No	495 (76.7)	424 (75.8)

Table S2: Nutritional profile of different cereal categories: median (min-max)

		n	Sugars (g/100g)	Fibre (g/100g)	Saturated fatty acids (g/100g)	Number of ingredients	Number of additives
Total sample		559	17.5 (0–40)	7.4 (0–29)	1.8 (0–16.4)	13 (1–62)	1 (0–7)
“Children’s” cereals	yes	161	24.8 (1.9–40)	4.8 (0–16)	1.2 (0–2.4)	14 (2–44)	0 (0–7)
	no	398	15.0 (0–34)	8.2 (1–29)	2.0 (0.1–4)	12 (1–62)	1 (0–7)
Muesli, Oats & other cereals flakes (MOCF)	yes	347	15.0 (0.5–34)	8.4 (1–29)	2.0 (0.2–16.4)	12 (1–62)	0 (0–7)
	no	212	22.4 (0–40)	5.3 (0–27)	1.2 (0–10.5)	14 (1–44)	1 (0–7)
“Organic” cereals	yes	307	14.0 (0–36)	8.2 (0–29)	1.8 (0.1–16.4)	11 (1–62)	0 (0–1)
	no	252	20.9 (0.7–40)	6.2 (1.2–27)	1.9 (0–12)	17 (1–46)	1 (0–7)

Table S3. Associations between the Initial Nutri-Score and cereal characteristics. Results profiles are reported in %

Initial Nutri-Score	Muesli, Oats & other cereals flakes (MOCF)		"Children's" product		"Organic" product	
	Yes	No	Yes	No	Yes	No
A	47.3	16.0	9.9	45.7	47.9	20.2
B	12.4	20.3	21.1	13.1	13.7	17.5
C	30.3	42.9	44.7	31.2	29.0	42.5
D	10.0	20.8	24.2	10.1	9.4	19.8
Chi-square	P <0.0001		P <0.0001		P <0.0001	
OR (CI95%)	0.33 (0.24–0.46)		3.60 (2.6–5.2)		0.35 (0.25–0.48)	

Table S4. Associations between the Modified Nutri-Score and cereal characteristics. Results profiles are reported in %

Modified Nutri-Score	Muesli, Oats & other cereals flakes (MOCF)		"Children's" product		"Organic" product	
	Yes	No	Yes	No	Yes	No
A	36.9	3.3	1.2	33.4	35.4	10.3
B	10.1	5.2	1.2	11.1	9.8	6.4
C	33.1	36.8	37.3	33.4	33.6	35.7
D	16.4	48.6	52.8	18.8	19.9	39.3
E	3.5	6.1	7.5	3.3	1.3	8.3
Chi-square	P <0.0001		P <0.0001		P <0.0001	
	0.17 (0.12–0.24)		6.50 (4.5–9.4)		0.26 (0.19–0.36)	
